# Does cognitive behavioral therapy alter mental defeat and cognitive flexibility in patients with panic disorder?

**DOI:** 10.1186/s13104-018-3130-2

**Published:** 2018-01-12

**Authors:** Shinobu Nagata, Yoichi Seki, Takayuki Shibuya, Mizue Yokoo, Tomokazu Murata, Yoichi Hiramatsu, Fuminori Yamada, Hanae Ibuki, Noriko Minamitani, Naoki Yoshinaga, Muga Kusunoki, Yasushi Inada, Nobuko Kawasoe, Soichiro Adachi, Keiko Oshiro, Daisuke Matsuzawa, Yoshiyuki Hirano, Kensuke Yoshimura, Michiko Nakazato, Masaomi Iyo, Akiko Nakagawa, Eiji Shimizu

**Affiliations:** 1 Department of Cognitive Behavioral Physiology, Graduate School of Medicine, Chiba University, Chiba, 1-8-1 Inohana, Chuo-ku, Chiba-shi, Chiba 260-8670 Japan; 2 Cognitive Behavioral Therapy Center, Chiba University Hospital, 1-8-1 Inohana, Chuo-ku, Chiba-shi, Chiba 260-8670 Japan; 3 Organization for Promotion of Tenure Truck, University of Miyazaki, 1-1, Gakuenkibanadai-Nishi, Miyazaki, 889-2192 Japan; 4 Inada Clinic, Osaka, 2-6-5 Johoku-cho, Takatsuki, Osaka 569-0071 Japan; 5 Clinic Adachi, Gifu, 62 Oikecho, Gifu, Gifu 500-8373 Japan; 6 United Graduate School of Child Development, Osaka University, Kanazawa University, Hamamatsu University School of Medicine, Chiba University and University of Fukui, Suita, Chiba University, 1-8-1 Inohana, Chuo-ku, Chiba-shi, Chiba 260-8670 Japan; 7 Department Psychiatry, Graduate School of Medicine, Chiba University, 1-8-1 Inohana, Chuo-ku, Chiba-shi, Chiba 260-8670 Japan; 8 Research Centre for Child Mental Development, Chiba University, 1-8-1 Inohana, Chuo-ku, Chiba-shi, Chiba 260-8670 Japan

**Keywords:** Cognitive behavioral therapy, Panic disorder, Mental defeat, Cognitive flexibility

## Abstract

**Objective:**

Mental defeat and cognitive flexibility have been studied as explanatory factors for depression and posttraumatic stress disorder. This study examined mental defeat and cognitive flexibility scores in patients with panic disorder (PD) before and after cognitive behavioral therapy (CBT), and compared them to those of a gender- and age-matched healthy control group.

**Results:**

Patients with PD (*n* = 15) received 16 weekly individual CBT sessions, and the control group (*n* = 35) received no treatment. Patients completed the Mental Defeat Scale and the Cognitive Flexibility Scale before the intervention, following eight CBT sessions, and following 16 CBT sessions, while the control group did so only prior to receiving CBT (baseline). The patients’ pre-CBT Mental Defeat and Cognitive Flexibility Scale scores were significantly higher on the Mental Defeat Scale and lower on the Cognitive Flexibility Scale than those of the control group participants were. In addition, the average Mental Defeat Scale scores of the patients decreased significantly, from 22.2 to 12.4, while their average Cognitive Flexibility Scale scores increased significantly, from 42.8 to 49.5. These results suggest that CBT can reduce mental defeat and increase cognitive flexibility in patients with PD

*Trial registration* The study was registered retrospectively in the national UMIN Clinical Trials Registry on June 10, 2016 (registration ID: UMIN000022693).

**Electronic supplementary material:**

The online version of this article (10.1186/s13104-018-3130-2) contains supplementary material, which is available to authorized users.

## Introduction

Panic disorder (PD) is a mental illness characterized by repeated panic attacks that exert a significant impact on daily functioning [1]. When PD symptoms intensify, anticipatory anxiety increases, making it difficult for individuals with the disorder to venture outside their homes. PD is estimated to be the 27th leading cause of nonfatal population burden [2]; the Japanese lifetime prevalence rate is .8% [3]. The Japanese comorbidity rate for PD and depression is approximately 50–60% [4]. Failure to seek PD treatment and the exacerbation of concomitant symptoms increase the likelihood of comorbidity with depression. The global prevalence rate for untreated PD has been estimated at 55.9% [5], highlighting the importance of implementing PD treatment strategies.

Cognitive behavioral therapy (CBT), both in isolation and in combination with pharmacotherapy, is one of the most effective PD treatment approaches [6]. Specifically, CBT’s effectiveness in treating PD has been shown to exceed both the placebo effect [7] and that of pharmacotherapy [8–10]. Although the concurrent use of CBT and medication is more effective than the use of either treatment separately during the acute phase, this difference in effectiveness declines over time [11–13].

Mental defeat is a thought process involving a loss of emotional autonomy and a sense of feeling broken or less than human, expressed through statements such as, “I feel like a loser.” It has been shown to contribute to posttraumatic stress disorder (PTSD) [14–17]. Responsiveness to CBT is lower in patients with chronic PTSD who have experienced mental defeat [14]. Moreover, chronic pain research has shown that the severity of mental defeat is associated with the secondary occurrence of psychosocial problems, including depression and anxiety [16, 17].

Cognitive flexibility is defined as the mental ability to switch between two different modes of thinking; it includes the ability to adapt one’s thoughts and actions in response to different situations [18]. Previous research has demonstrated that patients with anorexia nervosa [19] and PTSD [20] have less cognitive flexibility than healthy individuals. Research involving nonclinical samples has shown that cognitive flexibility is negatively associated with depression and anxiety [21]. Therefore, levels of cognitive flexibility may influence the severity of mental disorders.

To our knowledge, no previous studies have examined the effects of CBT on both mental defeat and cognitive flexibility in patients with PD. Therefore, the current study compared the effects of CBT on mental defeat and cognitive flexibility in patients with PD and healthy age- and gender-matched controls.

## Main text

### Methods

#### Participants and design

Participants were recruited through clinical referrals and web-based advertisements between April 2014 and March 2015. Fifteen patients with PD (13 women) participated in a single-arm, uncontrolled CBT trial registered in the National UMIN Clinical Trials Registry (ID: UMIN000022693) [22]. Participants met the criteria for PD according to the Diagnostic and Statistical Manual of Mental Disorders Fifth Edition [1] and scored ≥ 8 points on the Panic Disorder Severity Scale–Self Report (PDSS-SR) [23].

Thirty-five age- and gender-matched healthy controls (28 women; *M*_age_ = 42.5, *SD* = 10.3, range: 23–68 years) completed the Mini-International Neuropsychiatric Interview [24, 25]; none met the diagnostic criteria for mental disorders and all had PDSS-SR scores of ≤ 8 and Patient Health Questionnaire-9 (PHQ-9) scores of ≤ 10.

#### Measures

The Mental Defeat Scale (MDS) is a 24-item questionnaire [16, 26] that measures mental defeat, using a 5-point Likert scale. Total scores range from 0 to 96, with higher scores indicating more severe mental defeat. The Cognitive Flexibility Scale (CFS) is a 12-item questionnaire [27, 28] that measures cognitive flexibility using a 6-point Likert scale. Total scores range from 12 to 72, with higher scores indicating greater cognitive flexibility.

The PDSS-SR is a 7-item questionnaire that measures overall PD severity [23, 29, 30] using a 5-point Likert scale. Total scores range from 0 to 28, with higher scores indicating more severe PD symptoms. The Panic and Agoraphobia Scale is a 13-item questionnaire that measures PD symptom severity using a 5-point Likert scale [31, 32].

The PHQ-9 is a 9-item questionnaire that measures depression severity using a 4-point Likert scale [33, 34]. The Generalized Anxiety Disorder-7 scale is a 7-item questionnaire that measures the severity of generalized anxiety disorder using a 4-point Likert scale [35, 36]. The EuroQol-5D is a 5-item questionnaire that evaluates quality of life using a 3-point Likert scale [37, 38].

Patients with PD received 16 weekly, 50-min CBT sessions and completed all of the aforementioned questionnaires prior to CBT (baseline), mid-CBT (after 8 weeks), and post-CBT (after 16 weeks). The control group completed only the MDS, CFS, PDSS-SR, and PHQ-9 questionnaires prior to CBT (baseline).

#### CBT intervention

The CBT intervention focused on changes in the catastrophic misinterpretation of bodily sensations [39]. In addition, concepts relating to social anxiety disorder (SAD) in the Clark and Wells Model of Social Phobia were applied in the intervention [40], as PD has much in common with SAD [41–43]. After each session, patients completed homework, which enabled them to master new skills and use them in daily life. Eight clinical psychologists and two psychiatrists administered the CBT; the group was supervised on a weekly basis by a senior supervisor [44]. Further information regarding the CBT program is provided in Seki et al. [22].

The main treatment steps were as follows: (a) development of an individualized version of the cognitive-behavioral model of PD; (b) role-playing behavioral experiments with and without safety behaviors; (c) restructuring catastrophic self-imagery induced by bodily sensations; (d) practicing external focus and shifting attention; (e) behavioral experiments to assess negative catastrophic beliefs; (f) rescripting early memories associated with negative images in panic-related situations; (g) modification of problematic pre- and post-event processing; (h) discussing differences between participants’ beliefs and those of others; (i) coping with persistent assumptions; and (j) relapse prevention. Pre-CBT, mid-CBT, and post-CBT assessments were implemented prior to session (a), at the beginning of session (e), and following session (j).

#### Statistical analysis

Demographic data from patients with PD and control participants were compared prior to CBT. Continuous variables were compared using a *t* test, whereas categorical variables were compared using a Chi square test of association. Pre-CBT, mid-CBT, and post-CBT questionnaire scores were examined via a repeated-measures ANOVA. An ANOVA was performed to examine differences in the study variables between patients with PD and control participants. The post hoc analysis involved *t* tests with a Bonferroni correction; effect sizes were calculated using Cohen’s *d* [45]. Associations between the study variables were examined over time and between groups using the Pearson’s correlation coefficient. The significance level was set at *p* < .05. Data were analyzed using SPSS Version 23 (SPSS Inc., Chicago, IL, USA).

### Results

#### Demographic characteristics

There were no significant differences in demographic characteristics between the patients with PD and the control participants, except in the category of “employment status.” Patients with PD had a higher unemployment rate (Table 1).

**Table 1:** Participants’ demographic characteristics

Measure	Patients with PD (*n* = 15)	Control group (*n* = 35)	Statistics
Age	38.6 years (*SD* 9.6)	42.5 years (*SD* 10.3)	*t*(48) = − 1.47, *p* = .15
Gender	13 female, 2 male	28 female, 7 male	*χ*^*2*^(1) = .00, *p* = 1.00
Educational background			*χ*^*2*^(1) = 6.17, *p* = .46
High school	2	4	
< 3 years of college/university	8	7	
≥ 3 years of college/university	5	24	
Marital status			*χ*^*2*^(1) = 2.69, *p* = .26
Single	6	12	
Married	8	23	
Divorced	1	0	
Employment status			*χ*^*2*^(1) = 7.96, *p* = .02
Employed full time	5	19	
Part-time/homemaker	7	16	
Unemployed	3	0	
comorbid agoraphobia (M.I.N.I)	13		
Comorbid axis I diagnoses (M.I.N.I)			
No comorbid condition (PD only)	12		
Depression	1		
Other anxiety disorder	3		
Medication			
Benzodiazepine	11		
Antidepressant	9		
Benzodiazepine and antidepressant	8		
No medication	3		
Mental defeat			
MDS mean (*SD*)	22.2 (16.6)	5.4 (5.3)	
Cognitive flexibility			
CFS mean (*SD*)	42.8 (9.7)	52.6 (7.5)	
Associated psychopathology			
PDSS-SR mean (*SD*)	12.1 (4.0)	.1 (.4)	
PHQ-9 mean (*SD*)	8.0 (3.2)	2.6 (2.4)	

*CFS* Cognitive Flexibility Scale, *MDS* Mental Defeat Scale, *M.I.N.I*. Mini International Neuropsychiatric Interview, *PD* panic disorder, *PDSS-SR* Panic Disorder Severity Scale-Self Report, *PHQ-9* Patient Health Questionnaire-9

#### Patients with PD: pre- versus mid- versus post-CBT assessment

The average PDSS-SR and PHQ-9 scores of the patients with PD decreased significantly between the pre- and mid-CBT assessments, and between the pre- and post-CBT assessments (*p* < .05; Table 2).

**Table 2:** Outcome measures for each assessment point

Measures	Pre-CBT	Mid-CBT	Post-CBT	Statistic	Effect size (Cohen’s *d*)			Post hoc
	Mean (*SD*)	Mean (*SD*)	Mean (*SD*)		Pre- to mid-CBT	Mid- to post-CBT	Pre- to post-CBT	
Mental Defeat Scale								
MDS	22.2 (16.6)	14.5 (16.0)	12.4 (12.8)	*F*(2, 28) = 7.54, *p* < .01	.47	.15	.66	pre > mid pre > post
Cognitive flexibility								
CFS	42.8 (9.7)	49.4 (7.8)	49.5 (5.9)	*F*(2, 28) = 12.56, *p* < .01	.75	.02	.83	pre < mid pre < post
Associated psychopathology								
PDSS-SR	12.1 (4.0)	7.5 (3.3)	5.5 (3.5)	*F*(2, 28) = 19.04, *p* < .01	1.26	.59	1.77	pre > mid pre > post
PHQ-9	8.0 (3.2)	5.4 (2.5)	5.2 (3.1)	*F*(2, 28) = 6.48, *p* < .01	.91	.07	.89	pre > mid pre > post

*CBT* Cognitive behavioral therapy, *CFS* Cognitive Flexibility Scale, *MDS* Mental Defeat Scale, *PDSS-SR* Panic Disorder Severity Scale-Self Report, *PHQ-9* Patient Health Questionnaire-9

The average pre-, mid-, and post-CBT MDS scores of the patients with PD were 22.2 (*SD* 16.6), 14.5 (*SD* 16.0), and 12.4 (*SD* 12.8), respectively (Table 2). The repeated-measures ANOVA indicated a significant change in MDS scores over time, *F*(2, 28) = 7.54, *p* < .001. Post-hoc *t* tests indicated that the average MDS scores decreased significantly between the pre- and mid-CBT assessments (*d* = .47) and between the pre- and post-CBT assessments (*d* = .66).

The average pre-, mid-, and post-CBT CFS scores were 42.8 (*SD* 9.7), 49.4 (*SD* 7.8), and 49.5 (*SD* 5.9), respectively. The repeated-measures ANOVA indicated a significant change in CFS scores over time *F*(2, 28) = 12.56, *p* < .001. Post-hoc *t* tests indicated that the average CFS scores increased between the pre- and mid-CBT assessments (*d* = .75) and between the pre- and post-CBT assessments (*d* = .83).

#### Patients with PD versus control participants

**Fig. 1:**
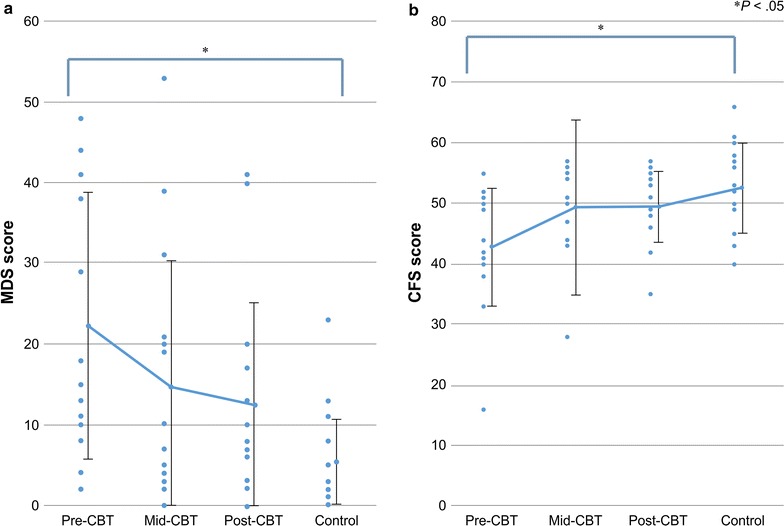
Comparison of Mental Defeat Scale (MDS) and Cognitive Flexibility Scale (CFS) scores between patients with panic disorder and control participants. The graph on the left **a** shows MDS scores and that on the right **b** shows CFS scores. Average values were calculated as arithmetic means, and error bars represent standard deviations

The ANOVA results showed that MDS scores differed significantly between the patients with PD and control participants, *F* (3, 76) = 7.52, *p* < .01. Post-hoc *t* tests indicated that the pre-CBT MDS scores of the patients with PD were significantly higher than those of the control participants (*p* < .05). Mid- and post-CBT scores did not differ significantly between the groups (Fig. 1).

Cognitive Flexibility Scale scores differed significantly between the patients with PD and the control participants, *F*(3, 76) = 5.62, *p* < .01. Post-hoc *t* tests showed that the pre-CBT CFS scores of the patients with PD were significantly lower than those of the control participants (*p* < .05). Mid- and post-CBT scores did not differ significantly between the groups (Fig. 1).

#### Correlations

Post-CBT MDS scores were significantly correlated with post-CBT CFS scores in both patients with PD (*r* = − .709, *p* < .01) and control participants (*r* = − .465, *p* < .01). No significant correlations were observed between the scores on GAD-7, PAS, or EuroQol-D5 in either group.

### Discussion

Our findings showed that MDS scores of patients with PD decreased and their CFS scores increased between the pre- and post-CBT assessments. This finding suggests that CBT can reduce mental defeat and increase cognitive flexibility in the patients with PD.

Although no previous studies have examined pre- and post-CBT MDS scores, several sessions implemented in the present study, including those involving safety behaviors and attentional bias, have been an effective part of CBT treatment for SAD [41–43]. These findings lend credibility to the use of CBT to treat mental defeat in patients with PD. By understanding the mechanisms underlying PD and the skills needed to manage symptoms, patients can reduce feelings of mental defeat, including helplessness and powerlessness.

Mid- and post-CBT scores did not differ significantly, perhaps because later sessions, including sessions (f) and (g), focused on the cognitive aspects of PD [41–43]; this may have stabilized reductions in mental defeat between the mid- and post-CBT assessments.

Cognitive behavioral therapy proved an effective treatment for enhancing cognitive flexibility, as it allowed patients to understand the mechanisms underlying PD and acquire skills to manage their symptoms. Consequently, patients were able to transform their initial catastrophic thoughts about panic symptoms (e.g., symptoms can kill) into objective thoughts (e.g., symptoms do not lead to death). This finding suggests that patients with PD, whose cognitive distortions have become entrenched, can develop flexibility in their thinking.

As shown in Additional file 1, the nonclinical MDS scores observed in previous studies, including those of individuals with chronic pain, were much higher than those observed in the present study. This discrepancy may reflect the differences in the research approach and context, including variations in data-collection conditions and cultural factors. For example, subclinical symptoms of depression were not excluded in Oshiro and Shimizu’s study [26]. Moreover, previous studies have reported a wide range of nonclinical CFS levels in various mental disorders [19, 20, 27, 28, 46, 47]. This variation may also reflect the research and contextual differences.

Significant correlations were observed between post-CBT MDS and CFS scores in both patients with PD and control participants, indicating that the MDS and CFS scores of patients with PD were similar to those of nonclinical populations following CBT.

### Conclusions

The present study revealed that, although patients with PD initially exhibit more intense mental defeat and lower levels of cognitive flexibility than control participants do, the CBT intervention reduces mental defeat and increases cognitive flexibility to levels observed in nonclinical populations.

## Limitations

The limitations of this study include the small sample size, gender disparity among participants, and the lack of follow-up assessment. The higher prevalence of PD in women than in men may explain the gender disparity [4]. The control group completed the questionnaires prior to CBT at baseline, therefore any impact of the passage of time on the measured outcomes was not evaluated. As the study did not include a randomized control group, it is unclear whether reductions in mental defeat and increases in cognitive flexibility resulted from CBT or from natural processes. Future research should involve randomized controlled trials with larger and more diverse samples, and should follow participants for a longer period. In addition, it is unclear which sessions led to the improvements observed. Future research should involve the administration of the MDS and CFS during all CBT sessions.

## Additional file

**Additional file 1.** Comparison of scores on the Mental Defeat Scale and the Cognitive Flexibility Scale.

## Abbreviations

CBT: cognitive behavioral therapy
CFS: Cognitive Flexibility Scale
MDS: Mental Defeat Scale
PD: panic disorder
PDSS-SR: Panic Disorder Severity Scale-Self Report
PHQ-9: Patient Health Questionnaire-9
PTSD: posttraumatic stress disorder
SAD: social anxiety disorder
