# Supplementary material for: Does cognitive behavioral therapy alter mental defeat and cognitive flexibility in patients with panic disorder?
Source: BMC Res Notes. 2018 Jan 12;11:23. doi: 10.1186/s13104-018-3130-2 (PMC5767061; doi:10.1186/s13104-018-3130-2)
Supplement: Supplementary file 1 — Additional file 1. Comparison of scores on the Mental Defeat Scale and the Cognitive Flexibility Scale. [file 13104_2018_3130_MOESM1_ESM.docx]

**Additional File 1** Comparison of the scores of the Mental Defeat Scale and the Cognitive Flexibility Scale

| **Scale** | **Study** | **Sample type** | **Country** | ***N*** | **Mean** | ***SD*** |
| --- | --- | --- | --- | --- | --- | --- |
| **MDS** | Tang et al. (2007) | Chronic pain | UK | 27 | 32.1 | 26.4 |
|  | Present study (pre-CBT) | Panic disorder | Japan | 15 | 22.2 | 16.6 |
|  | Present study (post-CBT) | Panic disorder | Japan | 15 | 12.4 | 12.8 |
|  | Tang et al. (2007) | Nonclinical | UK | 27 | 17.9 | 20.6 |
|  | Oshiro (unpublished observations submitted) | Nonclinical | Japan | 372 | 12.0 | 14.3 |
|  | Present study | Nonclinical | Japan | 35 | 5.4 | 4.3 |
| **CFS** | Palm et al. (2011) | PTSD | USA | 248 | 56.6 | 7.5 |
|  | Lounes et al. (2011) | Anorexia nervosa | UK | 45 | 45.4 | 9.6 |
|  | Johnco et al. (2014) | Depression and anxiety | Australia | 44 | 50.1 | 7.9 |
|  | Lee et al. (2014) | GAD | USA | 11 | 51.2 | 8.6 |
|  | Present study (pre-CBT) | Panic disorder | Japan | 15 | 42.8 | 9.7 |
|  | Present study (post-CBT) | Panic disorder | Japan | 15 | 49.5 | 5.9 |
|  | Martin et al. (1995) | Nonclinical | USA | 27 | 55.5 | 6.3 |
|  | Lounes et al. (2011) | Nonclinical | UK | 49 | 58.8 | 4.6 |
|  | Lee et al. (2014) | Nonclinical | USA | 11 | 61.1 | 4.5 |
|  | Oshiro et al. (2016) | Nonclinical | Japan | 335 | 46.3 | 7.7 |
|  | Present study | Nonclinical | Japan | 35 | 52.6 | 7.6 |

Abbreviations: MDS: Mental Defeat Scale; CFS: Cognitive Flexibility Scale; GAD: Generalized Anxiety Disorder; PTSD: Posttraumatic stress disorder
